# *Azadirachta indica* A. Juss Fruit Mesocarp and Epicarp Extracts Induce Antimicrobial and Antiproliferative Effects against Prostate (PC-3), Breast (MCF-7), and Colorectal Adenocarcinoma (Caco-2) Cancer Cell Lines through Upregulation of Proapoptotic Genes

**DOI:** 10.3390/plants11151990

**Published:** 2022-07-30

**Authors:** Omer H. M. Ibrahim, Magdi A. A. Mousa, Khalid A. Asiry, Nabil A. Alhakamy, Kamal A. M. Abo-Elyousr

**Affiliations:** 1 Department of Arid Land Agriculture, Faculty of Meteorology, Environment and Arid Land Agriculture, King Abdulaziz University, Jeddah 21589, Saudi Arabia; mamousa@kau.edu.sa (M.A.A.M.); kasiry@kau.edu.sa (K.A.A.); ka@kau.edu.sa (K.A.M.A.-E.); 2 Department of Pharmaceutics, Faculty of Pharmacy, King Abdulaziz University, Jeddah 21589, Saudi Arabia; nalhakamy@kau.edu.sa; 3 Center of Excellence for Drug Research and Pharmaceutical Industries, King Abdulaziz University, Jeddah 21589, Saudi Arabia; 4 Mohamed Saeed Tamer Chair for Pharmaceutical Industries, Faculty of Pharmacy, King Abdulaziz University, Jeddah 21589, Saudi Arabia

**Keywords:** antiproliferative effect, antimicrobial effect, colon cancer, breast cancer, prostate cancer, neem

## Abstract

Effective alternative strategies and methodological approaches are critically necessary for cancer prevention and therapy. In this study, we investigated the antitumor potential of neem fruit mesocarp and epicarp extracts. The chemical composition of the derived extracts was characterized using GC–MS. Data were collected on the antimicrobial activity of the extracts in addition to the cytotoxicity effect evaluated against PC-3, MCF-7, and Caco-2 cancer cell lines, compared with the normal Vero cells. Cell-cycle arrest, apoptosis, and expression of apoptosis-related genes were assessed on PC-3 cells. Both extracts had significant antiproliferative effects on all tested cell lines in a dose-dependent manner, with the mesocarp extract being more potent. Both extracts also showed high antibacterial and antifungal activities. These results were related to the chemical constituents of the extracts identified by the GC–MS analysis. The extract of neem fruit mesocarp caused cell-cycle arrest at G2/M phase of PC-3 cells. The cytotoxicity of neem mesocarp extract is strongly correlated with the induction of apoptosis, where it caused downregulation of the antiapoptotic BCL2 gene but upregulation of the proapoptotic P53 and BAX genes. This study showed that neem fruit extract is potential anticancer material in the future.

## 1. Introduction

Although cancer is considered one of the most common devastating diseases and is ranked as the second leading cause of death in humans, currently available cancer chemotherapeutic agents are not tissue-specific. This results in adverse effects on the host cells, thereby increasing morbidity. Disadvantages of monotargeted therapies including ineffectiveness, lack of safety, and excessive cost have encouraged many pharmaceutical companies to invest in the research of multitargeted therapies. Natural plant-based products provide solutions for all these disadvantages being multitargeting, nontoxic or less toxic, and readily available at low cost compared to synthetic agents [1,2,3,4]. The unexplored potential of the plant kingdom should be exploited for broad-spectrum natural anticancer remedies.

Neem is an omnipotent large evergreen tree and a sacred gift of nature. Neem tree is mainly cultivated in the Indian subcontinent in addition to the tropical zones of Africa and south Asia for ornamental, environmental, and medicinal purposes. It is a member of the mahogany family, Meliaceae, and known as *Azadirachta indica* A. Juss. For over 2000 years, neem has been used in traditional medicine in India to treat a range of ailments [5]. There is a long list of diseases and conditions affected by this, including fevers, tumors, strangulation of the intestines, measles, smallpox, cholera, diabetes, ulcers, malaria, gingivitis, and periodontitis, etc. [6]. Recently, several biological activities have been proved for various parts of neem tree including anti-inflammatory, antiulcer, antimalarial, antibacterial, and antioxidant activities [7], together with antiseptic, emollient, astringent, anthelmintic and insecticidal properties [8]. Components of this compound have also shown therapeutic implications for modulating cancer cell signaling pathways. Because of neem’s broader pharmacological properties, it might also be a promising candidate for preventing and treating tumors [9].

Several previous studies have determined the antioxidant, antimicrobial, and anticancer potential of neem extracts from different plant parts along with individual compounds commonly present in these extracts [10]. These include unripe and ripe fruits [11], whole fruit and flesh [12], fruit epicarp [13], leaves [14,15,16], flowers and stem bark [17], and roots [18]. Manikandan et al. [19] attributed the modulating capabilities of neem leaf extract for cancer cell proliferation and apoptosis to its antioxidant properties. Common phytochemicals in neem fruits, including fatty acids and their esters also including palmitic and oleic acids, showed antimicrobial and anticancer potential by several authors [20]. The aspects induced by several fatty acids include induction of cell-cycle arrest at G0/G1, inhibition of DNA topoisomerase, and induction of apoptosis and autophagy via blocking the Akt/mTOR pathway [21,22]. Neem fruit extract also contains 2,3-dihydro-3,5-dihydroxy-6-methyl-4H-pyranone (DDMP), a dihydropyranone proved to have strong antioxidant activity in glucose–histidine Maillard reaction products [11,23]. DDMP-induced apoptosis in colon cancer cells (SW620 and HCT116) via the modulation of the activity of NF-_K_B, where it suppressed the anti-apoptotic genes (BCL2), whereas it induced the expression of the apoptotic genes (BAX, cleaved caspase-3 and cleaved PARP) [24]. Despite the promising biological activity of neem’s unripe and ripe fruits, only a few studies have dealt with their proapoptotic effects and thus more in-depth studies are urged to help understand their specific anticipated activities against various cancer cell lines. 

Therefore, the main goal of the proposed project is to investigate the in vitro antitumor potential of neem fruit mesocarp extract against PC-3, MCF-7, and Caco-2 cell lines. The chemical composition of the extracts and their antimicrobial activities are provided. Cell-cycle arrest, cell apoptosis and necrosis and expression of apoptosis-related genes are assessed on PC-3 cells.

## 2. Materials and Methods

### 2.1. Preparation of Neem Fruit Extracts

Fruits were collected from a neem tree (*Azadirachta indica* A. Juss.) cultivated in the vicinity of Assiut University, Egypt. The tree and the samples collected therefrom were authenticated by the Department of Ornamental Plants and Landscape Gardening, Assiut University, Egypt. After cleaning the fruits with running water, fruit epicarp (epicarp) and fruit mesocarp (mesocarp) were separately shed dried, pulverized, and used for the preparation of the methanolic extracts by maceration in 80:20 methanol: water solvent at 1:10 *w/v* ratio of sample to solvent and kept under continuous shaking for three days at room temperature. The macerate was filtered, and the process was repeated two more times at three-day intervals. The filtrates were combined and concentrated using a rotary evaporator (Hidolph VV2000) and then freeze-dried using a Telstar-LyoQuest plus-55 lyophilizer. The yield of the extract was measured and stored in dark glass tubes at −20 °C for further analyses. 

### 2.2. Gas Chromatography–Mass Spectrometry (GC–MS) Analysis of Neem Extract

The chemical constituents of the studied neem extracts were identified by Trace GC-TSQ mass spectrometer (Thermo Scientific, Austin, TX, USA) supplemented with a capillary column TG-5MS (30 m × 0.25 mm × 0.25 µm film thickness). In the initial set-up, the oven temperature was 50 °C and was then raised gradually from 250 to 300 °C over a period of 2 min at 5 °C/min. A 270 °C temperature was set for the injector, while a 260 °C temperature was set for the MS transfer line. One milliliter per minute of helium was injected as the carrier gas. Autosampler ASI300 paired with GC in split mode was used to inject diluted samples automatically after a 4 min solvent delay. In full scan mode, ionization voltages of 70 eV were used to collect mass spectra over the range of *m/z* 50–650. A temperature of 200 °C was set for the ion source. Comparing the mass spectrum of each component with that of the mass spectral databases WILEY 09 and NIST 14 allowed identification of the components.

### 2.3. Antifungal Activity of Neem Extracts

Antifungal activity of the methanolic extracts of neem fruit mesocarp and epicarp was tested in vitro against different phytopathogenic fungi (*Rhizoctonia solani*, *Penicillium italicum,* and *Fusarium oxysporium*). To obtain extract concentrations of 15.6, 31.3, 62.5, 125, 250, 500, and 1000 ppm, different volumes of crude methanol extracts were added into PDA media before pouring into Petri plates, after which the plates were inoculated with 2 mm fungal plugs in the center and cultured for 10 days at 28 °C. For comparison, hymexazol at 1000 ppm was employed as a positive control. The linear growth was calculated for the treated plates (B) when the growth in the control treatment (A) covered the plats.

### 2.4. Antibacterial Activity of Neem Extracts

The antibacterial activity of the two neem fruit extracts was studied in vitro against *Agrobacterium tumefaciens, Serratia marcescens, and Acinetobacter johnsonii*. For the agar diffusion test, NSA medium was used according to Brulez and Zeller [25]. In brief, a suspension of different bacteria was spread over the agar surface and, after drying, different concentrations of the tested extracts (15.6, 31.3, 62.5, 125, 250, 500, and 1000 ppm) in addition to amoxicillin (62.5 ppm) as a positive control were pipetted into a 9 mm punch. Four replicates were used for each treatment. After 2 days of cultivation at 27 °C, inhibition zones in mm were measured for the control (A) and the treated plates (B) and the growth inhibition percentage was calculated according to Equation (1):

(1) $$\mathrm{Growth}\;\mathrm{inhibition}=\left(\frac{A-B}{A}\right)\times 100$$

### 2.5. Cytotoxicity Test

#### 2.5.1. Cell Cultures

Four cell lines were included in the current study, which were obtained from ATCC Company, USA. Three human cancer cell lines were tested, viz. the prostate (PC-3), breast (MCF-7), and colorectal adenocarcinoma (Caco-2), in addition to normal Vero cells (Accession number: ATCC CCL-81) isolated from kidney of *Cercopithecus aethiops*. MCF-7 cells (Accession number: ATCC HTB-22) were isolated from the mammary gland; breast-derived from pleural effusion metastases, PC-3 cells (Accession number: ATCC CRL-1435) were isolated from the prostate derived from bone metastases; and Caco-2 cells (Accession number: ATCC ATB-37) were isolated from colon tissues. The cells were grown on RPMI medium 1640 augmented with fetal bovine serum (10%), penicillin G (100 units/mL), and streptomycin sulfate (100 mg/mL). Incubation of the cultured cells was done in a CO_2_ incubator at 37 °C. Harvesting of the cells was performed by the addition of 0.25% of trypsin-0.025% of EDTA-2Na in PBS. 

#### 2.5.2. MTT Assay

Cytotoxic activity of neem extracts was investigated using MTT assay according to the method described by Bahuguna et al. [26] with some modifications. In brief, harvested cells of the tested cell lines were plated in the 96-well plates (1 × 10^5^ cells/mL (100 mL/well)) and incubated in a CO_2_ incubator at 37 °C. Incubation was done for 24 h to allow a monolayer sheet of cells to develop. The growth medium was decanted, and the cell layer was washed two times with a fresh growth medium. Two-fold dilutions (31.25 to 1000 ppm) of the extract samples were prepared using a maintenance medium (RPMI-1640 medium supplemented with 2% of fetal bovine serum) and used to treat the cells at different concentrations, while the control wells received only a maintenance medium. Doxorubicin was used as a positive control at the same concentrations prepared from the tested extracts. Visual observation was to record any physical signs of cell toxicity. After 48 h of incubation, the culture medium was decanted and 20 mL of MTT solution (5 mg/mL) was added to each well and incubated in dark for 4 h under the same conditions. The MTT was then removed and DMSO was added to solubilize the formazan crystal (MTT metabolic product), and incubated for 30 min under the same conditions. Using an ELISA reader, the data were collected on the optical densities of the plated cells at 560 nm wavelength. The cytotoxicity percentage was estimated from the formula: cytotoxicity percentage = (A5_60_ control − A5_60_ sample)/A5_60_ control × 100. The IC_50_ was calculated for the tested extracts against each carcinoma cell line as well as the normal Vero cells, from which the selectivity index (SI) was calculated as SI = IC_50_ calculated for normal cells/ IC_50_ calculated for cancer cells. 

### 2.6. Cell-Cycle Arrest Assessment 

PC-3 cells were used to test the cell-cycle distribution according to Alqahtani et al. [27] and Nasr et al. [28]. To perform the test, PC-3 cells were incubated for 24 h after being treated with the methanolic extract of neem fruit mesocarp (111 µg/mL). The collected cells from the control and treated cells were washed two times with cold PBS then fixed in cold 70% ethanol, and stored at 4 °C for 4 h. PBS was then used to rehydrate the fixed cells followed by incubation with RNase A at 100 μg/mL in order to degrade RNA, and propidium iodide at 100 μg/mL (ab139418_Propidium Iodide Flow Cytometry Kit/BD, Abcam, USA) for DNA staining. Flow cytometry (BD FACSCalibur) was used to determine the DNA content of the samples following 30 min of incubation. Propidium iodide fluorescence intensity was collected on FL_2_ of a flow cytometer and 488 nm laser excitation.

### 2.7. Flow Cytometry Assessment of Apoptotic vs. Necrotic Cells

The incidence of apoptosis and necrosis in the tested cancer cells was evaluated using an Apoptosis Detection Kit, FITC Annexin V with PI (BioVision, Milpitas, CA, USA). In accordance with the manufacturer’s instructions, the PC-3 cells were incubated in 6-well culture plates (4 × 10^5^ cells/well) for 24 h, and then treated with the methanolic extract of neem fruit mesocarp (111 µg/ mL) for the period of 24 h. After that, cells were collected and washed twice with PBS, and then resuspended in Annexin V-binding buffer (100 µL). Afterward, Annexin V-FITC and propidium iodide dyes at the rate of 5 μL each were mixed with the cells and incubated for 15 min under dark conditions. The flow cytometry (BD FACSCalibur) was used to assess the apoptotic vs. necrotic cell populations.

### 2.8. Assessment of Apoptosis-Related Gene Expression

Gene expression was assessed for the apoptosis-related genes (BAX, BCL2, and P53) using quantitative real-time PCR (qRT-PCR) analysis. PC-3 cells (4 × 10^5^ cells/mL) plated in a 6-well plate were exposed to neem extract at 111 μg/mL concentrations for 24 h. RNA was extracted using a TRIzol reagent from the treated and nontreated PC-3 cells. The extracted RNA at 1 μg was then converted to cDNA using a BioRad syber green PCR MMX kit, as per the manufacturer’s instructions. qRT-PCR was then conducted with specific primers of BAX, BCL2, and P53, as presented in Table 1, using the Rotorgene RT- PCR system (Corbett Research, Sydney, Australia) according to Nasr et al. [28]. 

### 2.9. Statistical Analysis 

All the experiments were carried out in three independent trials and data were presented as mean± standard deviation. Statistix software (ver. 8.1, Analytical Software, Tallahassee, FL, USA) was used to perform the statistical analysis. One-way ANOVA was applied with LSD to compare the group means. 

## 3. Results

### 3.1. Chemical Composition of Neem Fruit Mesocarp Extract

**Figure 1:**
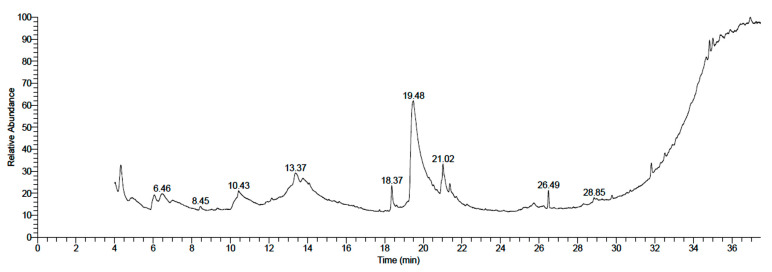
GC–MS chromatogram of methanolic fruit mesocarp extract of neem.

GC–MS analysis was applied to identify different active components of the methanolic neem extracts of fruit mesocarp (fruit mesocarp) and epicarp (fruit epicarp). In the case of neem fruit mesocarp, a total of eleven compounds were detected as displayed in Table 2 and Figure 1. D-Glucose, 4-O-α-D-glucopyranosyl- (maltose) represented the major constituent (45.19%) followed by moderate constituents, including oleic acid (10.1%) and octadecanoic acid, 2,3-dihydroxypropyl ester (9.51%), while the other components were represented in lower amounts. The identified components could be classified mainly as nonreducing disaccharide sugars, fatty acids, and fatty acid esters. 

**Figure 2:**
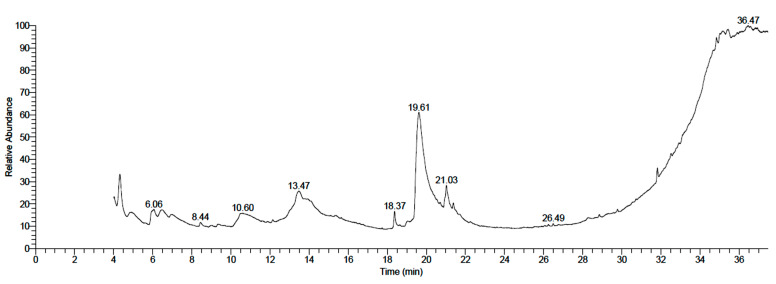
GC–MS chromatogram of methanolic fruit epicarp extract of neem.

On the other hand, 18 components were found in the fruit epicarp extract, as shown in Table 3 and Figure 2, in which d-Manno-l-gluco-octonic acid was the predominant component (43.64%). The other prevailing compounds included 4h-pyran-4-one, 2,3-dihydro-3,5-dihydroxy-6-methyl- (8.07%), Ethyl iso-allocholate (7.23%), Docosanoic acid, 1,2,3-propanetriyl ester (7.13%), Desulfosinigrin (6.04%), Oleic acid (5.86%), and Octadecanoic acid, 3-hydroxy-, methyl ester (4.80). 

### 3.2. Antimicrobial Activity of Neem Extracts

Both methanolic extracts of neem fruit mesocarp and epicarp showed a significant zone of inhibition against the human pathogenic bacterium Gram-positive *Acinetobacter johnsonii*, the bioagent bacterium Gram-positive *Serratia marcescens,* and the plant pathogenic bacterium Gram-negative *Agrobacterium tumefaciens* (Table 4). Growth inhibition of the three bacteria started with the concentration of 62.5 µg/mL for both extracts, which increased in a significant manner as the extract concentration was increased, reaching the maximum inhibition with the highest concentration (1000 µg/mL). Overall, fruit epicarp extract showed more antibacterial efficacy against the three bacteria compared to fruit mesocarp. Relative to the antibiotic treatment (Amoxicillin), *Acinetobacter johnsonii* was the most affected bacterium by both extracts. The maximum growth inhibitions recorded in response to fruit epicarp extract reached 211.11, 238.89, and 216.67%, which correspond approximately to 36, 45, and 42% of the inhibition induced by the antibiotic treatment. In the case of fruit mesocarp extract, the maximum growth inhibitions recorded were 172.2, 188.9, and 150.0%, which correspond approximately to 30, 35, and 29% induced by the antibiotic treatment, respectively.

Potential antifungal activity was exhibited by both extracts against *Rhizoctonia solani, Penicillium italicum,* and *Fusarium oxysporium,* as presented in Table 5. The growth of the tested fungi varied significantly in a concentration-dependent manner. The highest inhibition of fungal growth was obtained by the highest extract concentration (1000 µg/mL). Epicarp extract induced higher growth inhibition compared to the mesocarp extract against *Rhizoctonia solani*. Meanwhile, the mesocarp extract was more efficient against *Penicillium italicum* and *Fusarium oxysporium*. The highest growth inhibition (54.07%) was recorded with the mesocarp extract against *Penicillium italicum* compared to 68.15% obtained by the hymexazol treatment.

### 3.3. Cytotoxic Activity of Neem Extracts

**Figure 3:**
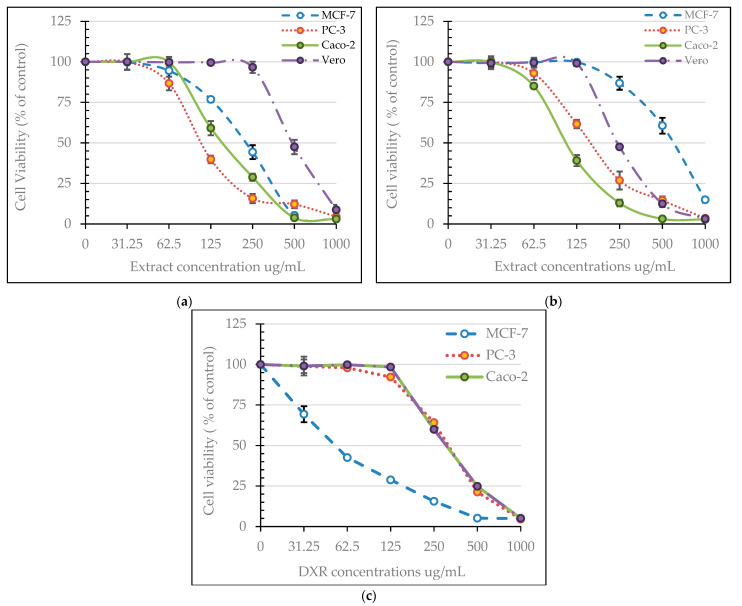
MTT assay results of viability/cytotoxicity on MCF-7, PC-3, Caco-2, and Vero cells: (**a**) effect of the methanolic extracts from neem fruit mesocarp; (**b**) effect of the methanolic extracts from neem fruit epicarp; (**c**) effect of doxorubicin. Cytotoxicity levels were derived from three experiments done in triplicate. Values are represented as the mean ± SD.

**Figure 4:**
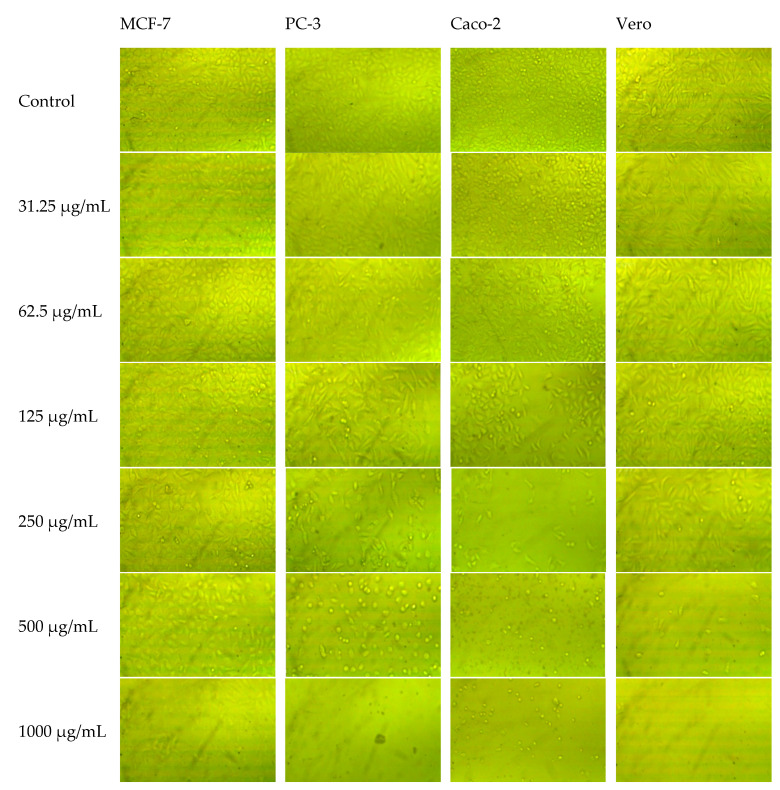
Morphology of MCF-7, PC-3, Caco-2, and Vero cells after 48 h of the treatment with different concentrations of the methanolic extract of neem fruit epicarp.

**Figure 5:**
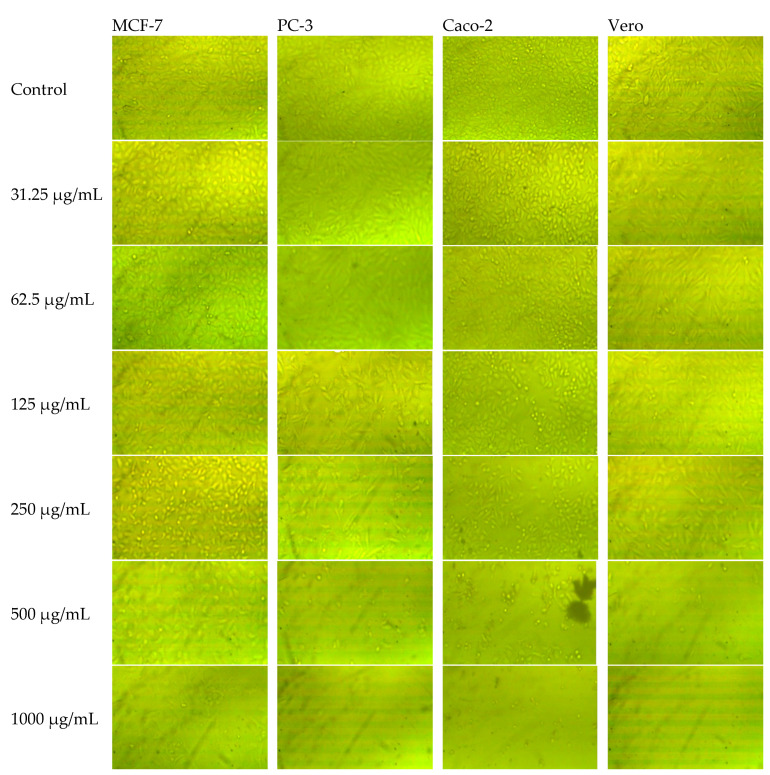
Morphology of MCF-7, PC-3, Caco-2, and Vero cells after 48 h from the treatment with different concentrations of the methanolic extract of neem fruit mesocarp.

The methanolic extracts of neem fruit epicarp and mesocarp persuaded a corresponding concentration-dependent inhibition of cell viability of the prostate (PC-3), breast (MCF-7), and colorectal adenocarcinoma (Caco-2), in addition to normal Vero cells (Figure 3). Changes in cell morphology induced by the increased concentrations of tested extracts are illustrated in Figure 4 and Figure 5. The calculated IC_50_ and selectivity index (SI) pointed out a clear variation between the cytotoxic effect of both extracts and the response of the three cell lines (Table 6). The mesocarp was more potent against the three cell lines exhibiting lower IC_50_ and thus higher selectivity index than the epicarp extract. Compared to doxorubicin, both extracts were more potent against Caco-2 and PC-3 cells. Caco-2 cell line was the most affected by the epicarp extract (IC_50_ µg/mL = 110.16, SI = 2.77), while PC-3 was the most affected line by the mesocarp extract (IC_50_ µg/mL = 111.76 µg/mL, SI = 5.26). Therefore, PC-3 cells treated with the mesocarp extract at 111 µg/mL were chosen to study the cell-cycle arrest, apoptosis, and necrosis, and the expression of apoptosis-related genes.

### 3.4. Effect of Neem Extract on Cell-Cycle Arrest in PC-3 Cells

**Figure 6:**
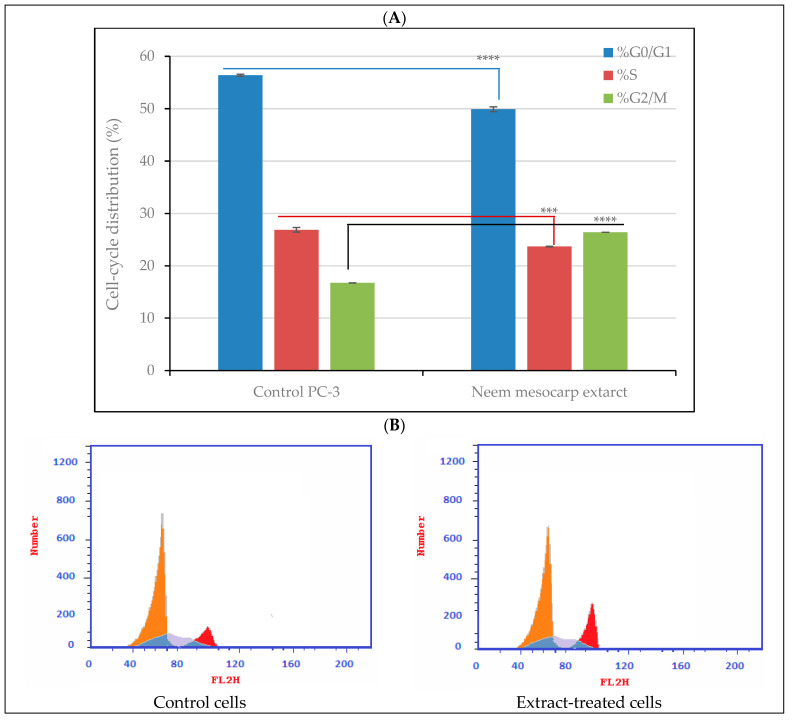
Cell-cycle distribution of PC-3 cells treated with methanolic fruit mesocarp extracts of neem at 111 µg/mL: (**A**) quantitative cell-cycle distribution % illustrated by the bar graph with cell growth arrest@ G2/M; (**B**) flow cytometry histogram showing DNA content of neem extract-treated cells. Significance differences between treated and control cells were determined using unpaired *t*-test, *** *p* < 0.001, and **** *p* < 0.0001. The data presented are the means of three replicates; ±SD indicated by the vertical bars.

The flow cytometric assay indicated that the methanolic extract of neem fruit mesocarp caused cell-cycle arrest at G2/M phase of treated PC-3 cells (Figure 6). The percentage of G2/M phase reached 26.41% in treated cells compared to 16.74% in the control cells. This was accompanied by fewer cells in the proportion of other phases compared with the control. 

### 3.5. Effect of Neem Extract on Apoptosis and Necrosis of Cells

**Figure 7:**
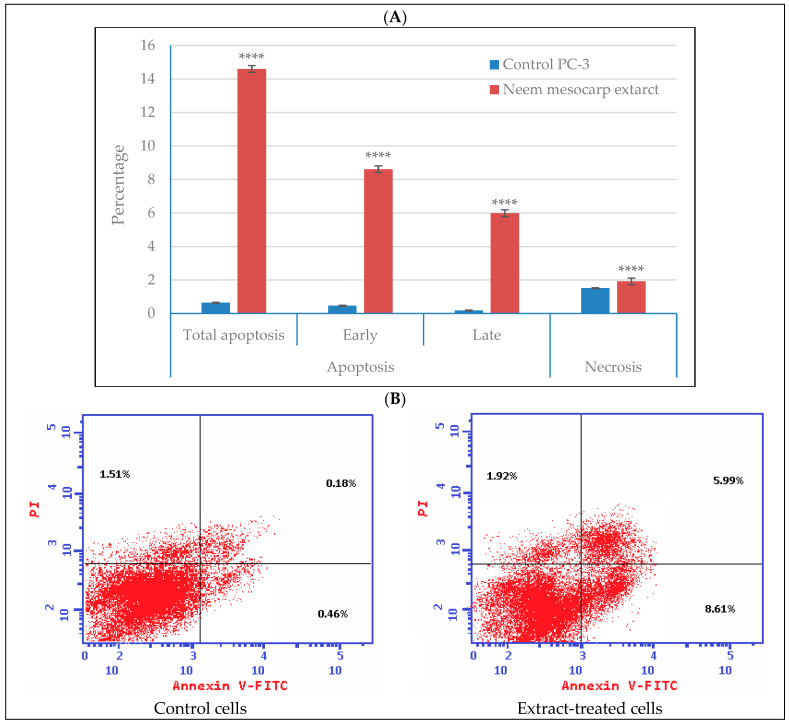
Apoptotic effect of methanolic fruit mesocarp extracts of neem at 111 µg/mL on PC-3 cells: (**A**) quantitative cell-cycle distribution % illustrated by the bar graph with cell growth arrest@ G2/M; (**B**) flow cytometry dot plots of extract-treated cells showing necrotic cells (upper left quadrant), late apoptotic cells (upper right quadrant), viable cells (lower left quadrant), and early apoptotic cells (lower right quadrant). Significance differences between treated and control cells were determined using unpaired *t*-test, **** *p* < 0.0001. The data presented are the means of three replicates; ±SD indicated by the vertical bars.

Analysis of apoptosis and necrosis in PC-3 cells indicated that the treatment with neem mesocarp extract increased the amount of total apoptosis to 16.5%, the early apoptosis to 8.6%, and the late apoptosis to 6.0%, compared with 2.15, 0.46, and 0.18% in the control, respectively (Figure 7). The necrosis, on the other hand, recorded a slight increase in the treated cells (1.9%) compared with that in the control (1.51%). These results suggest that the cytotoxicity of neem mesocarp extract is strongly correlated with the induction of apoptosis.

### 3.6. Effect of Neem Extract on the Expression of Apoptosis-Related Genes in PC-3 Cells

**Figure 8:**
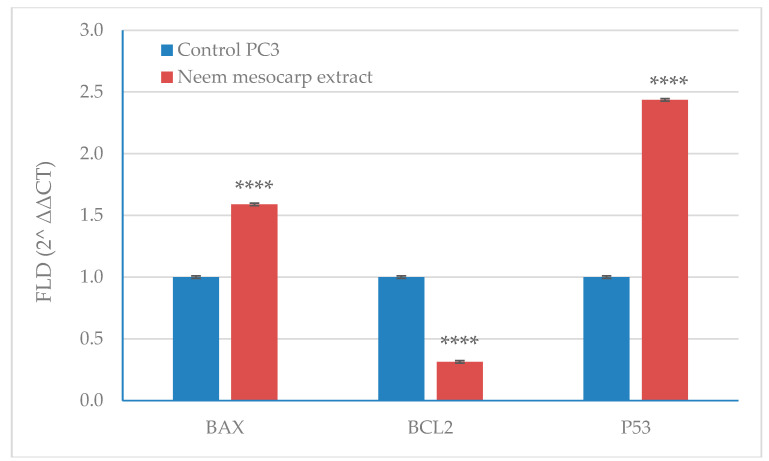
qRT-PCR quantification of apoptosis-related genes expression (BAX, BCL2, and P53) in PC-3 cells treated with the methanolic extract of neem fruit mesocarp at 111 µg/mL, compared with untreated cells. Significance differences between treated and control cells were determined using unpaired *t*-test, **** *p* < 0.0001. The data presented are the means of three replicates; ±SD indicated by the vertical bars.

The data illustrated in Figure 6 display the qRT-PCR quantification of apoptosis-related gene expression (BAX, BCL2, and P53) in PC-3 cells treated with the methanolic extract of neem fruit mesocarp at 111 µg/mL, compared with the untreated cells. The treated cells showed a clear decrease in the expression of the antiapoptotic BCL2 gene (30% of the control), but both P53 and BAX were upregulated (Figure 8). Downregulation of BCL2 and upregulation of P53 and BAX genes facilitate the explanation of the mechanism by which neem extract induced apoptosis.

## 4. Discussion

Natural plant-based products provide solutions for the disadvantages of chemotherapeutic agents—multitargeting, nontoxic or less toxic, and readily available at low cost, compared to synthetic agents [1,2]. Broad-spectrum natural anticancer remedies should be extended through systematic screening of the unexplored wealth of the plant kingdom. The neem plant has attracted the attention of researchers for its potent antioxidant, antimicrobial, and anticancer activities of extracts from different plant parts, including unripe and ripe fruits [11], whole fruit and flesh [12], fruit epicarp [13], leaves [14,15,16], flowers and stem bark [17], and roots [18]. Corresponding to these studies, the current work also revealed that the methanolic extracts of neem fruit mesocarp and epicarp had significant antiproliferative effects on all tested cell lines (PC-3, MCF-7, and Caco-2) in a dose-dependent manner, with the mesocarp extract being more potent against the three cell lines. PC-3 was the most affected line by the mesocarp extract, exhibiting the highest selectivity index (5.26). Both extracts also showed high antibacterial and antifungal activities when tested against the human pathogenic bacterium Gram-positive *Acinetobacter johnsonii*, the bioagent bacterium Gram-positive *Serratia marcescens,* and the plant pathogenic bacterium Gram-negative *Agrobacterium tumefaciens*, along with three phytopathogenic fungi, i.e., *Rhizoctonia solani, Penicillium italicum,* and *Fusarium oxysporium*. To fully understand the effect of these extracts, it is worth identifying their active constituents and presenting their biological activities from previous literature. 

The GC–MS analysis of the two neem fruit extracts showed the presence of many phytochemicals with evident biological activities, which could be related to their antimicrobial and antiproliferative activities. In a comparison of the identified constituents of both extracts, six common components were detected. These components included desulfosinigrin (5.98, 6.04%); ethyl iso-allocholate (6.08, 7.23%); hexadecanoic acid (5.84, 2.65%); oleic acid (10.06, 5.86%); á-D-Glucopyranose, 4-O-á-D-galactopyranosyl-(5.7, 2.6%), and 9,12,15-octadecatrienoic acid, 2,3-bis[(trimethylsilyl)oxy]propyl ester, (Z,Z,Z)-(2.9, 0.64%) in neem fruit mesocarp and epicarp extracts, respectively. Desulfosinigrin is a hydrolysis product of glucosinolates, which have been proven to play a role in reducing cancer risk. The inhibitory activity of desulfosinigrin was observed toward cyclin-dependent kinase by Krishnaveni [29], upon which he deduced the potential of desulfosinigrin as a therapeutic drug for cancer treatment. α-D-Glucopyranose, 4-O-α-D-galactopyranosyl- is a sugar moiety compound known for its preservative attributes [30]. The anticancer property may also be due to the presence of ethyl isoallocholate, a steroidal derivative reported for its potent anticancer property, especially against A549 lung cancer cells [31]. Anticancer activities of fatty acids and their esters have been discussed by several authors [20]. Of the fatty acids detected in both extracts, 9,12,15-octadecatrienoic acid, 2,3-bis[(trimethylsilyl)oxy]propyl ester, (Z,Z,Z)- has antimicrobial, anticancer, hepatoprotective, antiarthritic, antiasthma, and diuretic effects [32]. The potential in vitro anticancer activity of hexadecanoic acid (palmitic acid) was recorded by Bharath et al. [21] with cell-cycle arrest observed at the G0/G1 phase. Harada et al. [33] also reported the cytotoxicity of palmitic acid isolated from red seaweed, *Amphiroa zonata*, toward human leukemic cells, where it inhibited DNA topoisomerase I. This indicated that palmitic acid inhibited the proliferation of HDF cells without affecting normal cells. Jiang et al. [22] revealed the potent anticancer effect of oleic acid in tongue squamous cell carcinoma by inducing apoptosis and autophagy via blocking the Akt/mTOR pathway and cell-cycle arrest at G0/G1. The evident activities of these components in previous literature could help justify their antimicrobial and anticancer activities.

The neem fruit mesocarp extract was characterized by the presence of d-Glucose, 4-O-à-D-glucopyranosyl- (Maltose); octadecanoic acid, 2,3-dihydroxypropyl ester; 1,2-benzenedicarboxylic acid, bis(2-ethylhexyl) ester; hexadecanoic acid 2,3-dihydroxypropyl ester; and 12-Octadecadienoic acid (Z,Z)-. Most of these have been reported as potent bioactive components. The spectrum of antibacterial activity was ascribed by several authors to the presence of hexadecanoic acid, 2,3-dihydroxypropyl ester [34] and 1,2-benzenedicarboxylic acid, bis(2-ethylhexyl) ester [35]. The other components include octadecanoic acid, 2,3-dihydroxypropyl ester (Stearin), a glycerol derivative, which has anticancer and antimicrobial activities [36], and 9,12-Octadecadienoic acid (Z,Z)-, which is a fatty acid reported to have anti-inflammatory, cancer-preventive, and antiprostatitic properties [30]. However, fruit epicarp extract comprised a higher number of constituents, of which d-Manno-l-gluco-octonic acid, a high carbon sugar, represented the main component. The other components included 2,3-dihydro-3,5-dihydroxy-6-methyl-4H-pyranone (DDMP), a dihydropyranone proved to have strong antioxidant activity in glucose–histidine Maillard reaction products [23], which has been detected previously in the methanolic extract of ripe neem seed by Guchhait et al. [11]. DDMP-induced apoptosis in colon cancer cells (SW620 and HCT116) via the modulation of the activity of NF-_K_B, where it suppressed the antiapoptotic genes (BCL2), whereas it induced the expression of the apoptotic genes (BAX, cleaved caspase-3, and cleaved PARP) [24]. Other fatty acid esters were also detected, such as docosanoic acid, 1,2,3-propanetriyl, which has the property of antioxidant and hypocholesterolemic activities [37]. 

To develop a new anticancer drug, it is of high importance to inspect the regulatory mechanism of cell growth and apoptosis cells as an essential cell-death process, which is considered a potential pathway for the development of new drugs [38,39]. It is clear from the effect of the methanolic extract of neem fruit mesocarp on the cell cycle of treated PC-3 cells causing arrest at G2/M phase. Inspection of apoptosis and necrosis of cells revealed that the cytotoxicity of neem mesocarp extract is strongly correlated with the induction of apoptosis. Additionally, the treated cells showed a clear downregulation of the antiapoptotic BCL2 gene compared with the upregulation of the proapoptotic P53 and BAX genes. Our data are in line with several previous studies that reported the antiproliferative, antibacterial, and antioxidant effects of neem fruit extract and its components. Guchhait et al. [11] revealed that the methanolic extract of neem unripe and ripe seeds showed antibacterial activity against *Staphylococcus aureus* (Gram-positive) than *Vibrio cholerae* (Gram-negative) and anticancer activity against the normal blood lymphocytes, and the breast cancer cell line (MDA-MB-231). Natarajan et al. [40] showed high antimicrobial activity of neem seed extract against various dermatophytes. 

## 5. Conclusions

The methanolic extracts of neem fruit mesocarp and epicarp had significant antiproliferative effects on all tested cell lines (PC-3, MCF-7, and Caco-2) in a dose-dependent manner, with the mesocarp extract being more potent against the three cell lines. Both extracts also showed high antibacterial and antifungal activities. The GC–MS analysis of the two neem fruit extracts showed the presence of many phytochemicals with evident biological activities, which could be related to their antimicrobial and antiproliferative activities. The methanolic extract of neem fruit mesocarp caused cell-cycle arrest at G2/M phase of treated PC-3 cells. The cytotoxicity of neem mesocarp extract is strongly correlated with the induction of apoptosis, where it caused downregulation of the antiapoptotic BCL2 gene but upregulation of the proapoptotic P53 and BAX genes.

## Figures and Tables

**Table 1:** Specific primers of BAX, BCL2, and P53 used to perform qRT-PCR analysis for PC-3 cells in response to neem extract treatment.

Gene	Primer
BAX	F: 5’-ATGGACGGGTCCGGGGAG-3’
	R: 5’-ATCCAGCCCAACAGCCGC-3’
BCL2	F: 5’-AAG CCG GCG ACGACT TCT-3’
	R: 5’-GGT GCC GGT TCA GGTACT CA-3’
p53	F: 5’-ATGTTTTGCCAACTGGCCAAG -3’
	R: 5’-TGAGCAGCGCTCATGGTG-3’

**Table 2:** Chemical composition of methanolic fruit mesocarp extract of neem screened by GC–MS.

No.	Compound	RT	Area %	Formula	MW	CAS Number
1	Octadecanoic acid, 2,3-dihydroxypropyl ester	4.32	9.51	C_21_H_42_O_4_	358	123-94-4
2	α-D-Glucopyranose, 4-O-α-D-galactopyranosyl-	6.04	5.70	C_12_H_22_O_11_	342	5965-66-2
3	Desulfosinigrin	13.36	5.98	C_10_H_17_NO_6_S	279	5115-81-1
4	Hexadecanoic acid	18.36	5.84	C_16_H_32_O_2_	256	57-10-3
5	Maltose	19.43	45.19	C_12_H_22_O_11_	342	69-79-4
6	9,12-Octadecadienoic acid (Z,Z)-	20.94	1.83	C_18_H_32_O_2_	280	60-33-3
7	Oleic Acid	21.02	10.06	C_18_H_34_O_2_	282	112-80-1
8	Hexadecanoic acid, 2,3-dihydroxypropyl ester	21.38	3.18	C_19_H_38_O_4_	330	542-44-9
9	1,2-benzenedicarboxylic acid, bis(2-ethylhexyl) ester	26.49	3.74	C_24_H_38_O_4_	390	117-81-7
10	9,12,15-octadecatrienoic acid, 2,3-bis[(trimethylsilyl)oxy]propyl ester, (Z,Z,Z)-	31.82	2.90	C_27_H_52_O_4_Si_2_	496	55521-22-7
11	Ethyl iso-allocholate	34.84	6.08	C_26_H_44_O_5_	436	NA

**Table 3:** Chemical composition of methanolic fruit epicarp extract of neem screened by GC–MS.

No.	Compound	RT	Area %	Formula	MW	CAS Number
1	4h-pyran-4-one, 2,3-dihydro-3,5-dihydroxy-6-methyl-	4.32	8.07	C_6_H_8_O_4_	144	28564-83-2
2	2-amino-5-guanidino-pentanoic acid	4.83	0.82	C_6_H_14_N_4_O_2_	174	74-79-3
3	3,4,5,6tetrahydroxy-2-oxohexanoic Acid	4.91	1.88	C_6_H_10_O_7_	194	NA
4	Octadecanoic acid, 3-hydroxy-, methyl ester	5.94	4.80	C_19_H_38_O_3_	314	2420-36-2
5	α-d-glucopyranose, 4-o-α-d-galactopyranosyl-	6.47	2.61	C_12_H_22_O_11_	342	5965-66-2
6	L-gala-l-ido-octonic lactone	8.44	0.88	C_8_H_14_O_8_	238	NA
7	Digitoxin	10.48	2.26	C_41_H_64_O_13_	764	71-63-6
8	Desulfosinigrin	13.45	6.04	C_10_H_17_NO_6_S	279	5115-81-1
9	Hexadecanoic acid	18.37	2.65	C_16_H_32_O_2_	256	57-10-3
10	d-Manno-l-gluco-octonic acid	19.59	43.64	C_8_H_16_O_9_	256	NA
11	[1,1’-bicyclopropyl]-2-octanoic acid, 2’-hexyl-, methyl ester	20.95	1.09	C_21_H_38_O_2_	322	56687-68-4
12	Oleic acid	21.03	5.86	C_18_H_34_O_2_	282	112-80-1
13	Ethyl iso-allocholate	29.78	7.23	C_26_H_44_O_5_	436	NA
14	9,12,15-octadecatrienoic acid, 2,3-bis[(trimethylsilyl)oxy]propyl ester, (Z,Z,Z)-	32.91	0.64	C_27_H_52_O_4_Si_2_	496	55521-22-7
15	Docosanoic acid, 1,2,3-propanetriyl ester	33.10	7.13	C_69_H_134_O_6_	1058	18641-57-1
16	Oleic acid, eicosyl ester	34.29	0.32	C_38_H_74_O_2_	562	22393-88-0
17	9-octadecenoic acid, 1,2,3-propanetriyl ester, (E,E,E)-	34.66	2.35	C_57_H_104_O_6_	884	537-39-3
18	Psi.,.psi.-carotene, 1,2-dihydro-1-hydroxy-	36.37	1.71	C_40_H_58_O	554	105-92-0

**Table 4:** Antibacterial activities of methanol extract of neem fruit epicarp (epicarp) against *Serratia marcescens, Acinetobacter johnsonii,* and *Agrobacterium tumefaciens*.

Extract	Concentrations (µg/mL)	Inhibition Zone (mm)			Growth Inhibition (%)		
		*Seratia*	*Acinetobacter*	*Agrobacterium*	*Seratia*	*Acinetobacter*	*Agrobacterium*
epicarp	15.6	6.0 h	6.0 h	6.0 h	0.00	0.00	0.00
	31.3	6.0 h	6.0 h	6.0 h	0.00	0.00	0.00
	62.5	6.7 gh	7.0 gh	7.7 g	11.11	16.67	27.78
	125	7.7 fg	8.3 fg	8.0 g	27.78	38.89	33.33
	250	12.7 de	13.7 de	13.7 de	111.11	127.78	127.78
	500	15.7 c	17.3 c	17.0 c	161.11	188.89	183.33
	1000	18.7 b	20.3 b	18.8 b	211.11	238.89	216.67
mesocarp	15.6	6.0 h	6.0 h	6.0 h	0.00	0.00	0.00
	31.3	6.0 h	6.0 h	6.0 h	0.00	0.00	0.00
	62.5	7.3 gh	7.3 gh	7.3 gh	22.22	22.22	22.22
	125	9.0 f	9.3 f	8.7 g	50.00	55.56	44.44
	250	11.7 e	12.0 e	11.3 f	94.44	100.00	88.89
	500	13.3 d	14.0 d	13.0 e	122.22	133.33	116.67
	1000	16.3 c	17.3 c	14.8 d	172.22	188.89	150.00
Negative control		6.0 h	6.00 f	6.0 h	0.00	0.00	0.00
Amoxicillin (62.5 ppm)		41.3 a	38.33 a	37.0 a	588.89	538.89	516.67

Values in the same column followed by different letters indicate significant differences among treatments according to the LSD test (*p* = 0.05).

**Table 5:** Antifungal activities of methanol extract of neem fruit epicarp (epicarp) against *Rhizoctonia solani, Penicillium italicum,* and *Fusarium oxysporium*.

Extract	Concentrations (µg/mL)	Inhibition Zone (mm)			Growth Inhibition (%)		
		*Rhizoctonia*	*Penicillium*	*Fusarium*	*Rhizoctonia*	*Penicillium*	*Fusarium*
epicarp	15.6	5.8 e	8.5 b	6.6 bc	35.19	5.93	26.67
	31.3	5.8 e	8.3 bc	6.6 b	35.56	7.78	26.30
	62.5	5.5 f	8.0 bcd	6.4 cd	38.52	10.74	28.89
	125	5.4 fg	7.5 ef	6.3 de	39.63	17.04	30.37
	250	5.1 h	7.5 ef	6.0 f	43.70	16.67	32.96
	500	4.4 i	7.0 f	5.8 gh	50.74	21.85	35.93
	1000	4.2 j	6.4 g	5.0 ij	53.70	28.89	44.07
mesocarp	15.6	6.7 b	7.9 cde	6.6 b	25.56	12.59	26.30
	31.3	6.6 bc	7.7 de	6.1 ef	27.04	14.44	31.85
	62.5	6.4 c	7.5 ef	5.9 fg	29.26	16.67	33.70
	125	6.1 d	6.2 gh	5.7 h	31.85	31.48	36.67
	250	6.0 de	5.9 h	5.2 i	33.33	34.07	41.85
	500	5.5 f	5.4 i	5.1 ij	38.89	39.63	42.96
	1000	5.3 gh	4.2 j	4.9 j	41.85	54.07	45.19
Negative control		9.0 a	9.0 a	9.0 a	0.00	0.00	0.00
Hymexazol (1000 ppm)		1.8 k	2.9 k	1.6 k	79.63	68.15	81.85

Values in the same column followed by different letters indicate significant differences among treatments according to the LSD test (*p* = 0.05).

**Table 6:** IC_50_ (µg/mL) and selectivity index of the methanolic extracts of neem fruit epicarp and mesocarp against different cell lines.

Neem Methanolic Extract	IC_50_ (µg/mL)				Selectivity Index		
	Vero	MCF-7	PC-3	Caco-2	MCF-7	PC-3	Caco-2
Fruit epicarp	305.40	629.23	176.31	110.16	0.49	1.73	2.77
Fruit mesocarp	588.30	228.86	111.76	180.77	2.57	5.26	3.25
Doxorubicin	35.09	5.40	34.11	35.09	6.50	1.03	1.00

## Data Availability

Not applicable.
